# Expanded safety analysis of pevonedistat, a first-in-class NEDD8-activating enzyme inhibitor, in patients with acute myeloid leukemia and myelodysplastic syndromes

**DOI:** 10.1038/bcj.2017.1

**Published:** 2017-02-03

**Authors:** R T Swords, J Watts, H P Erba, J K Altman, M Maris, F Anwer, Z Hua, H Stein, H Faessel, F Sedarati, B J Dezube, F J Giles, B C Medeiros, D J DeAngelo

**Affiliations:** 1 Leukemia Program, Sylvester Comprehensive Cancer Center, Miami, FL, USA; 2 Division of Hematology/Oncology, University of Michigan, Ann Arbor, MI, USA; 3 Northwestern Medicine Developmental Therapeutics Institute, Northwestern University, Chicago, IL, USA; 4 Colorado Blood Cancer Institute, Denver, CO, USA; 5 University of Arizona Cancer Center, Tucson, AZ, USA; 6 T Takeda Pharmaceuticals International Co., Cambridge, MA, USA; 7 Division of Hematology, Stanford University School of Medicine, Stanford, CA, USA; 8 Department of Medical Oncology, Dana-Farber Cancer Institute, Boston, MA, USA

Dear Sir,

We previously reported the therapeutic potential of pevonedistat (TAK-924/MLN4924), a novel inhibitor of protein neddylation, in patients with acute myeloid leukemia (AML).^1^ Pevonedistat is a small-molecule inhibitor of the neural cell developmentally downregulated 8 (NEDD8)-activating enzyme, which processes NEDD8 for binding to target substrates.^2, 3, 4, 5^ The most characterized NEDD8 target in cells are the Cullin-RING E3 ubiquitin ligases (CRLs), which direct the degradation of specific substrates through the proteasome (for example, p27, CDT1, Nrf-2).^6^ In the absence of NEDD8, CRL substrates accumulate causing antiproliferative effects in AML.^1^ In a phase I trial (C15003 study), patients with relapsed/refractory myelodysplastic syndrome or AML were treated with 1-h infusions of pevonedistat every 21 or 28 days across five schedules. On Schedule A, dose escalation commenced at 25 mg/m^2^ on Days 1, 3 and 5 in a standard ‘3+3' manner to determine the maimum tolerated dose (MTD). Following this, four additional schedules were evaluated with pevonedistat: Schedule B (Days 1, 4, 8 and 11 starting at 147 mg/m^2^); Schedule C (Days 1, 8 and 15 starting one dose level lower than the MTD determined for Schedule A (44 mg/m^2^)); Schedule D (Days 1, 3 and 5 stating at 45 mg/m^2^ to be combined with azacitidine 75 mg/m^2^ on Days 1–5 and 8–9); and Schedule E (Days 1, 3 and 5 at fixed dose of 50 mg/m^2^). Schedules A–C and E were administered every 21 days, whereas schedule D was administered every 28 days. Adverse events (AEs) on Schedules A and B were taken into account when designing Schedules C–E; based on Schedule A MTD, an expansion dose of 50 mg/m^2^ was selected for Schedule E. Results for Schedules A and B were published by Swords *et al.*,^7^ and the reported MTDs were 59 and 83 mg/m^2^, respectively. Hepatotoxicity was dose limiting for Schedule A and multiorgan failure (MOF) for Schedule B. The objective response rate in patients treated at or below the MTD was 17% for Schedule A and 10% for Schedule B, confirming single agent activity in refractory AML patients.^8^ Considering the promising safety and activity of pevonedistat reported thus far,^8, 9, 10^ we carried out a comprehensive safety analysis of all 72 patients treated across five schedules (A–E) in the C15003 study.

Seventy-two patients were evaluable for toxicity: Schedule A (*n*=27), Schedule B (*n*=26), Schedule C (*n*=2), Schedule D (*n*=1), and Schedule E (*n*=16). Most patients had AML (*N*=66, 92%), 5 had myelodysplastic syndrome (7%) and 1 patient had ALL (1%). Median age was 65.5 years (range: 19–84). The most frequently reported all-grade AEs were: pyrexia (49%), diarrhea (43%), decreased appetite (35%), peripheral edema (33%), dyspnea and fatigue (each 32%), febrile neutropenia (31%) and nausea (31%). These were generally mild and transient. The most common reported laboratory toxicities were elevations in aspartate aminotransferase (25%) and alanine transaminase (24%). Treatment-related myelosuppression was infrequent with thrombocytopenia reported in 18% of patients. Fifteen (21%) patients discontinued the study owing to AEs (Table 1). Overall, 56 (78%) patients experienced at least 1 serious adverse event (SAE): Schedule A=20 (74%), Schedule B=20 (77%), Schedule C=2 (100%), Schedule D=1 (100%), and Schedule E=13 (81%). The most common SAE overall was febrile neutropenia reported in 17 (24%) patients (A=8, B=6 and E=3). Other SAEs occurring in >2 patients were pneumonia (*n*=12, 17%), disease progression (*n*=9, 13%), fever (*n*=6, 8%), sepsis (*n*=6, 8%), intracranial hemorrhage (*n*=5, 7%), hypotension (*n*=4, 6%), gastrointestinal hemorrhage (*n*=3, 4%) and mental status changes (*n*=3, 4%). There were 28 (39%) on-study deaths and 4 of these deaths were due to sepsis (1 patient on Schedule A at 78 mg/m^2^), MOF (2 patients on Schedule B at 110 mg/m^2^ and 147 mg/m^2^) and cardiopulmonary failure related to leukastasis (1 patient on Schedule D at 45 mg/m^2^). Eighteen deaths, including three of the four deaths due to MOF/cardiopulmonary failure occurred during Cycle 1. The on-study deaths unrelated to pevonedistat (*n*=24) occurred owing to underlying disease (*n*=11), sepsis (*n*=3), intracranial hemorrhage (*n*=5), pneumonia (*n*=3), and 1 case each of pulmonary hemorrhage and pancytopenia.

Overall, there were 10 dose-limiting toxicities (DLTs) in 6 patients: transaminase elevation (Schedule A, 78 mg/m^2^); orthostatic hypotension (Schedule B, 110 mg/m^2^); cardiac failure (Schedule B, 147 mg/m^2^); MOF (Schedule B, 147 mg/m^2^); rash (Schedule E, 50 mg/m^2^); and elevated alanine transaminase (Schedule E, 50 mg/m^2^). With the exception of 1 DLT, all events occurred within 2 days of first exposure to pevonedistat. There were no DLTs at doses <50 mg/m^2^, and all Grade 4 DLTs occurred at the highest dose of pevonedistat administered (147 mg/m^2^). Mean pevonedistat plasma concentration–time profiles obtained on Day 1 of Cycle 1 (and Cycle 3 on Schedule E) are shown in Figure 1. No obvious differences in exposures were observed between Cycles 1 and 3, suggesting that pevonedistat PK remains stationary over time. Of the 55 response evaluable patients, there were 2 complete remissions (4%) on Schedule A, and 5 partial remissions (9%) across Schedule A (*n*=2), Schedule B (*n*=2) and Schedule E (*n*=1). The overall complete remission/partial remission rate was 13%. Thirty-four patients (62%) had stable disease after treatment with ⩾4 courses of pevonedistat (Schedule A=14, Schedule B=13, Schedule E=7). All responses were in patients with AML.

The principal serious and potentially drug-related toxicities noted in this trial were hepatotoxicity and sepsis syndromes with MOF. The exact mechanisms accounting for the hepatotoxicity observed in some patients treated with pevonedistat is unclear. *In vitro* RNA interference to disrupt NEDD8 conjugation induces shape changes in HEK293 and HeLa cells (edema and swelling). This effect may relate to the accumulation of the cullin-dependent GTPase RhoA, which regulates cytoskeleton proteins.^11^ This effect is reproducible with pevonedistat treatment *in vitro* and is reversible by RhoA knockdown. It is interesting to speculate that transient leakage of transaminases from edematous hepatocytes in treated patients may have accounted for some of the laboratory toxicities observed in this study. A more compelling perspective on pevonedistat-induced liver toxicity was presented by Wolenski *et al.*,^12^ who demonstrated that treatment with pevonedistat lowered the activation threshold for tumor necrosis factor (TNF)-mediated cell death, by conferring cellular sensitivity to low concentrations of TNF-α. Patients with higher circulating levels of TNF-α (typically the case during sepsis) treated with pevonedistat in our study may have been more prone to the tissue-damaging effects of this cytokine. This mechanism may have contributed to the MOF events observed. It should be noted that MOF was rare in this trial and commonly occurred in heavily pretreated AML patients treated at higher doses of pevonedistat (other than the 1 patient on Schedule D (45 mg/m^2^) who died of MOF from leukostasis, there were no MOF events at doses of pevonedistat ⩽83 mg/m^2^). In order to study potential links with cytokine signaling and systemic inflammatory syndromes, we measured plasma cytokines (interleukin-1β and TNF-α) in treated patients. All the samples from patients treated on Schedule E were below the detectable limits of the assay. None of these patients had MOF events. Cytokine data were not available for patients who had developed MOF (Schedules A and B), which was a limitation of our study.

The most common AE reported was pyrexia (*n*=35, 49%) and pneumonia was the most common infection (*n*=15, 21%). Establishing the contribution of pevonedistat to these toxicities is challenging. However, there have been a number of reports describing the effects of impaired protein neddylation on innate immunity. For example, the perforin family of pore-forming proteins (the perforins), key bactericidal effector molecules of the innate immune system, require ubiquitin side chains for function. McCormack *et al.*^13^ demonstrated that direct small interfering RNA knockdown of CRLs reduced bactericidal activity of perforin proteins by blocking their ubiquitination. The same phenotype was achieved with pharmacological inhibition of CRLs using pevonedistat. Mice treated with pevonedistat and subsequently inoculated with salmonella died faster from sepsis and organ failure, compared with untreated controls. Despite these interesting observations, none of the patients with MOF on this study had documented bacterial infections. Others have shown that the antigen-presenting ability of dendritic cells and macrophages is markedly impaired by disrupting the neddylation of target substrates either genetically or chemically.^14^ This effect suggests a potential use for pevonedistat in the management of autoimmune disorders and graft versus host disease. Elucidation of the immunomodulatory effects of pevonedistat will help to further define both the promise and limitation of his new agent. In summary, the most serious toxicity was observed at higher doses of pevonedistat. For this reason, doses beyond 100 mg/m^2^ are not being considered for further investigation and no cases of drug-related MOF have been noted at doses currently in development. Pevonedistat combination studies for patients with myeloid malignancies are ongoing.^9, 15^

## Figures and Tables

**Figure 1:**
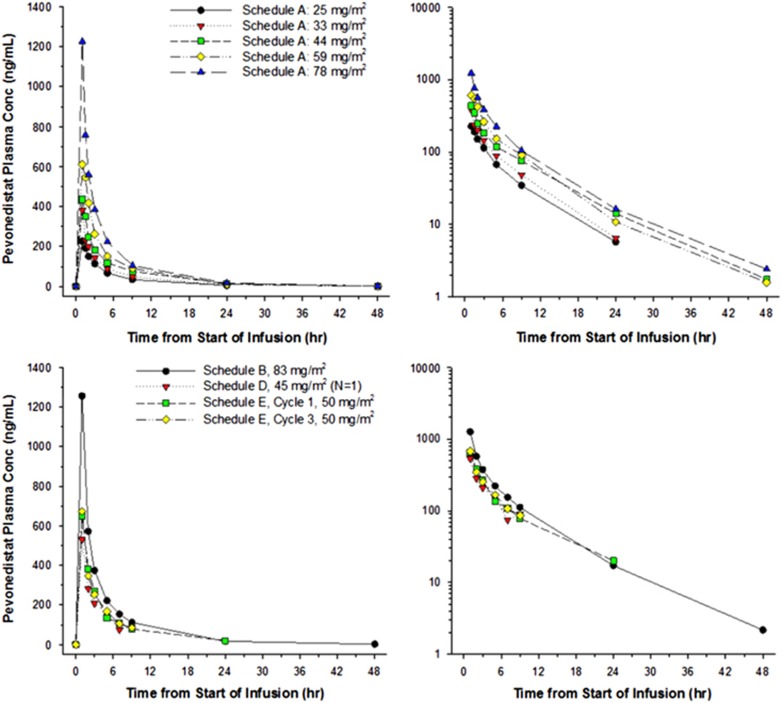
Mean pevonedistat plasma concentration–time profiles. Serial samples were obtained on Day 1 of Cycle 1 (and Day 1 of Cycle 3 on Schedule E) following a 1-h intravenous infusion and presented graphically (linear and semi-log plots, left and right panels, respectively).

**Table 1:** Treatment-emergent adverse events (AE)

*AE,* n *(%)*	*Schedule A*^a^ (n=*27)*	*Schedule B (*n=*26)*	*Schedule C (*n=*2)*	*Schedule D (*n=*1)*	*Schedule E (*n=*16)*	*Total (*n=*72)*
*Any AE*^b^	27 (100)	26 (100)	2 (100)	1 (100)	16 (100)	72 (100)
Pyrexia	12 (44)	16 (62)	0	0	7 (44)	35 (49)
Diarrhea	14 (52)	9 (35)	2 (100)	0	6 (38)	31 (43)
Anorexia	11 (41)	7 (27)	1 (50)	0	6 (38)	25 (35)
Peripheral edema	11 (41)	6 (23)	1 (50)	0	6 (38)	24 (33)
Fatigue	9 (33)	9 (35)	0	0	5 (31)	23 (32)
Dyspnea	6 (22)	10 (38)	0	1 (100)	6 (38)	23 (32)
FN	11 (41)	8 (31)	0	0	3 (19)	22 (31)
Nausea	10 (37)	7 (27)	0	0	5 (31)	22 (31)
Chills	8 (30)	11 (42)	0	0	1 (6)	20 (28)
Dizziness	11 (41)	4 (15)	1 (50)	0	4 (25)	20 (28)
Myalgia	8 (30)	7 (27)	1 (50)	0	4 (25)	20 (28)
Increased AST	9 (33)	3 (12)	1 (50)	0	5 (31)	18 (25)
Increased ALT	8 (30)	4 (15)	1 (50)	0	4 (25)	17 (24)
Headache	5 (19)	7 (27)	1 (50)	0	4 (25)	17 (24)
Epistaxis	6 (22)	6 (23)	0	0	5 (31)	17 (24)
Vomiting	8 (30)	5 (19)	1 (50)	0	2 (13)	16 (22)
Cough	7 (26)	6 (23)	0	0	2 (13)	15 (21)
Pneumonia	5 (19)	4 (15)	2 (100)	0	4 (25)	15 (21)
*Any treatment-related grade ⩾3 AE*^c^	16 (59)	20 (77)	2 (100)	1 (100)	12 (75)	51 (71)
Low platelets	2 (7)	2 (8)	0	0	0	4 (6)
MI	0	1 (4)	0	1 (100)	0	2 (3)
FN^d^	0	2 (8)	0	0	0	2 (3)
Increased AST	2 (7)	0	0	0	0	2 (3)
Increased ALT	1 (4)	0	0	0	1 (6)	2 (3)
Increased bilirubin	0	1 (4)	0	0	1 (6)	2 (3)
Hypoxia	1 (4)	1 (4)	0	0	0	2 (3)
Hypotension	1 (4)	1 (4)	0	0	0	2 (3)
MOF	0	2 (8)	0	0	0	2 (3)
Fatigue	2 (7)	0	0	0	0	2 (3)

Abbreviations: ALT, alanine transaminase; AST, aspartate aminotransferase; FN, febrile neutropenial; MI, myocardial infarction; MOF, multiorgan failure.

a Schdeule A (dose escalation cohort, Days 1, 3, 5); Schedule B (dose escalation cohort, Days 1, 4, 8, 11); Schedule C (weekly dosing schedule); Schedule D (combination with azacitidine); Schedule E (dose expansion cohort, Days 1, 3, 5).

b Any AE occurring in >20% of patients overall.

c Any treatment-related grade **⩾**3 AE occurring in >1 patient overall.

d Bacteremia occurred in a total of 3 patients on study (1 on Schedule A and 2 on Schedule E) (4%) and septic shock in 1 patient on Schedule C (1%); these events were reported as not related to pevonedistat.
